# Identification of Anhydrodebromoaplysiatoxin as a Dichotomic Autophagy Inhibitor

**DOI:** 10.3390/md21010046

**Published:** 2023-01-10

**Authors:** Limin Feng, Chung-Kuang Lu, Jiajun Wu, Leo Lai Chan, Jianbo Yue

**Affiliations:** 1 Shenzhen Key Laboratory in Sustainable Use of Marine Biodiversity, Research Centre for the Oceans and Human Health, City University of Hong Kong Shenzhen Research Institute, Shenzhen 518000, China; 2 National Research Institute of Chinese Medicine, Ministry of Health and Welfare, Taipei 11221, Taiwan; 3 Department of Bioscience and Institute of Genomics, National Yang Ming University, Taipei 11221, Taiwan; 4 State Key Laboratory of Marine Pollution, City University of Hong Kong, Hong Kong SAR 999077, China; 5 Department of Biomedical Science, City University of Hong Kong, Hong Kong SAR 999077, China; 6 Division of Natural and Applied Sciences, Synear Molecular Biology Lab, Duke Kunshan University, Kunshan 215316, China

**Keywords:** anhydrodebromoaplysiatoxin, autophagy, mTOR/p70S6K/FoxO3a, lysosome

## Abstract

Dysfunctional autophagy is associated with various human diseases, e.g., cancer. The discovery of small molecules modulating autophagy with therapeutic potential could be significant. To this end, we screened the ability of a series of metabolites isolated from marine microorganisms to modulate autophagy. Anhydrodebromoaplysiatoxin (ADAT), a metabolite yielded by the marine red algae *Gracilaria coronopifolia*, inhibited autophagosome-lysosome fusion in mammalian cells, thereby inducing the accumulation of autophagosomes. Treatment of cells with ADAT alkalinized lysosomal pH. Interestingly, ADAT also activated the mTOR/p70S6K/FoxO3a signaling pathway, likely leading to the inhibition of autophagy induction. ADAT had little effect on apoptosis. Our results suggest that ADAT is a dichotomic autophagy inhibitor that inhibits both late-stage (autophagosome-lysosome fusion) and early-stage (autophagy induction) autophagy.

## 1. Introduction

Macroautophagy (herein referred to as autophagy) is a cellular catabolic process for the degradation and/or recycling of protein aggregates, long-lived proteins, and damaged organelles to maintain cellular homeostasis [1]. Autophagy plays an important role in various cellular processes, such as cell growth, development, proliferation, and differentiation. Dysfunction of the autophagic process has been associated with the onset and development of many human diseases, including cancer, neurodegeneration, cardiovascular diseases, aging, metabolic disorders, and viral infection [2,3,4]. For many years, it was thought that antitumor agents might kill cells by inducing “autophagic cell death”. However, the process of autophagy has been found to be over-activated in many kinds of cancers [5,6], which enables cancer cells to survive radiation or chemotherapy [7]. It is thought that autophagy activation represents a cellular attempt to cope with stress induced by cytotoxic agents. Therefore, inhibition of autophagy (rather than induction of autophagy) might be beneficial in cancer treatment. Although many autophagy inhibitors have been identified, few have emerged with therapeutic potential against cancers. The high toxicity of most available autophagy inhibitors limits their clinical applications. Thus, the discovery of low-toxic autophagy inhibitors with therapeutic potential would be of great significance. Identifying specific and potent autophagy inhibitors is necessary to discover promising anticancer therapeutics.

Due to the harsh living environment (such as high-salinity, high-pressure, lightless, low temperature, hypoxia, etc.), marine organisms have unique metabolic and genetic pathways that produce metabolites with particular chemical structures and physiological activities. Many marine natural products and their secondary microbial metabolites have shown bioactivity, and they might become a rich resource for new drug research and development. Studies have shown that biological components or secondary metabolites from sponges, sea urchins, marine algae, bacteria, and fungi can be used as autophagy inducers or inhibitors [8]. For example, bafilomycins, purified from *Stretomyces* spp. in marine environments, are potent autophagy inhibitors, inducing autophagosome accumulation [9]. Sponge alkaloid manzamine A, similar to bafilomycin A1, also inhibited the autophagosome-lysosome fusion, resulting in a significant increase in the levels of autophagy markers, LC3-II and SQSTM1 [10].

Aplysiatoxins (ATXs) have been isolated from the cyanobacterium *Lyngbya majuscule* and are known to have tumor-promoting and protein kinase C (PKC) activation properties [11,12,13,14,15]. However, new ATX derivatives, such as Oscillatoxin I and debromoaplysiatoxin (DAT), have been reported to possess antiproliferative effects in cancer cells [16,17]. Anhydrodebromoaplysiatoxin (ADAT), one of the ATX-related compounds, known as a poisonous metabolite yielded by marine red algae *Gracilaria coronopifolia* [18] or marine cyanobacterium *Trichodesmium erythraeum* [19], is a potential PKC activator [20]. Here we showed that ADAT is a late-stage autophagy inhibitor, manifested by its ability to induce the accumulation of autophagosomes. Interestingly, ADAT also activated the mTOR/p70S6K/FoxO3a signaling pathway, likely leading to the inhibition of autophagy induction.

## 2. Results

### 2.1. ADAT Inhibited Cell Proliferation of HeLa or A549 Cells without Inducing Apoptosis

**Figure 1:**
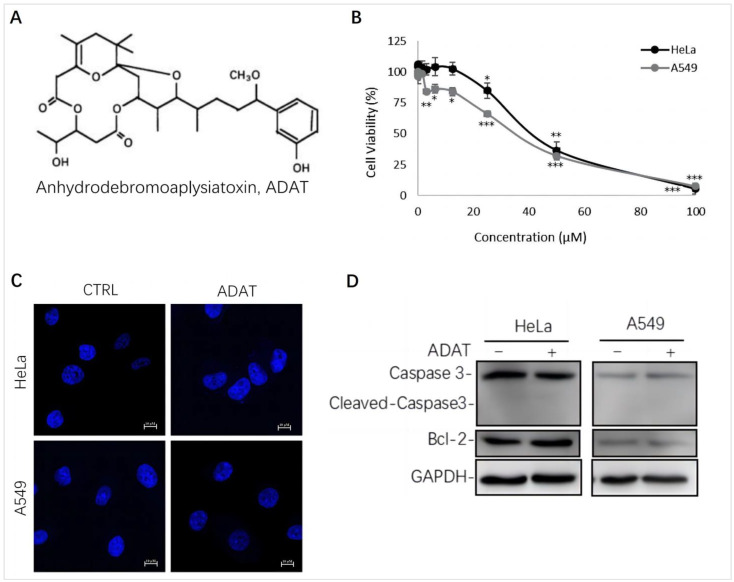
ADAT inhibits cell proliferation in HeLa or A549 cells. (**A**) Structure of ADAT. (**B**) Cell viability of HeLa and A549 cells after being treated with ADAT for 24 h. (**C**) 24 h treatment of ADAT (10 μM) did not induce apoptosis. Scale bar = 10 μm. (**D**) Treatment of cells with ADAT (10 μM) for 24 h did not induce the expression of cleaved-Caspase 3 and Bcl-2. All experiments were independently repeated three times (*n* = 3; *, *p* < 0.05; **, *p* < 0.01; ***, *p* < 0.001 vs. control).

We assessed the cytotoxicity of ADAT (Figure 1A) by using the MTT assay in HeLa cells (human cervical cancer cells) or A549 cells (human alveolar adenocarcinoma basal epithelial cells). We showed that the half maximal inhibitory concentration (IC50) of ADAT in HeLa or A549 cells was 36.64 μM or 32.23 μM, respectively (Figure 1B). Of note, a high concentration of ADAT (≥25 μM) inhibited the proliferation of HeLa and A549 cells after 24 h, while a low dose of ADAT (<12.5 μM) exhibited no cytotoxic activity in these cells (Figure 1B). As mentioned before, high toxicity of most available autophagy inhibitors limits their clinical applications, and low-toxic autophagy inhibitors with therapeutic potential would be meaningful. Taken together, we suggest that a low dose of ADAT might be suitable for the further assessment of its ability to modulate autophagy.

It is known that autophagy and apoptosis are key factors regulating the occurrence and development of cancer. Generally, high doses of autophagy inhibitors lead to high toxicity and make them useless in clinical practice. To assess whether ADAT at a dose with potential clinical use affects the apoptosis of HeLa or A549 cells, we performed DAPI staining and Western blot analysis of the expression of some apoptosis-related factors in ADAT-treated cells. The DAPI staining result showed that ADAT (10 μM) treatment did not have a significant effect on the nuclei of HeLa or A549 cells (Figure 1C). Likewise, the Western blot result showed that ADAT (10 μM) did not change the expression of Bcl-2 (a primary apoptosis regulator) and the activated form of Caspase 3 (cleaved-caspase-3) after 24 h treatment of in HeLa or A549 cells (Figure 1D). These results suggest that apoptosis was not the primary function of ADAT at low dose.

### 2.2. ADAT Is a Novel Autophagy Modulator

LC3-II is usually recruited to phagophores and remains on the inner membrane of the autophagosome until it is degraded in the autolysosome. SQSTM1 is a cargo receptor for the autophagic degradation of ubiquitinated substrates [21]. The level of LC3-II and SQSTM1 has been widely used as a marker to monitor autophagy accordingly. To identify novel autophagy modulators, we screened a series of natural products obtained from marine microorganisms in GFP and mRFP tandem-tagged LC3-expressing HeLa or A549 cells (tf-LC3B cells).

**Figure 2:**
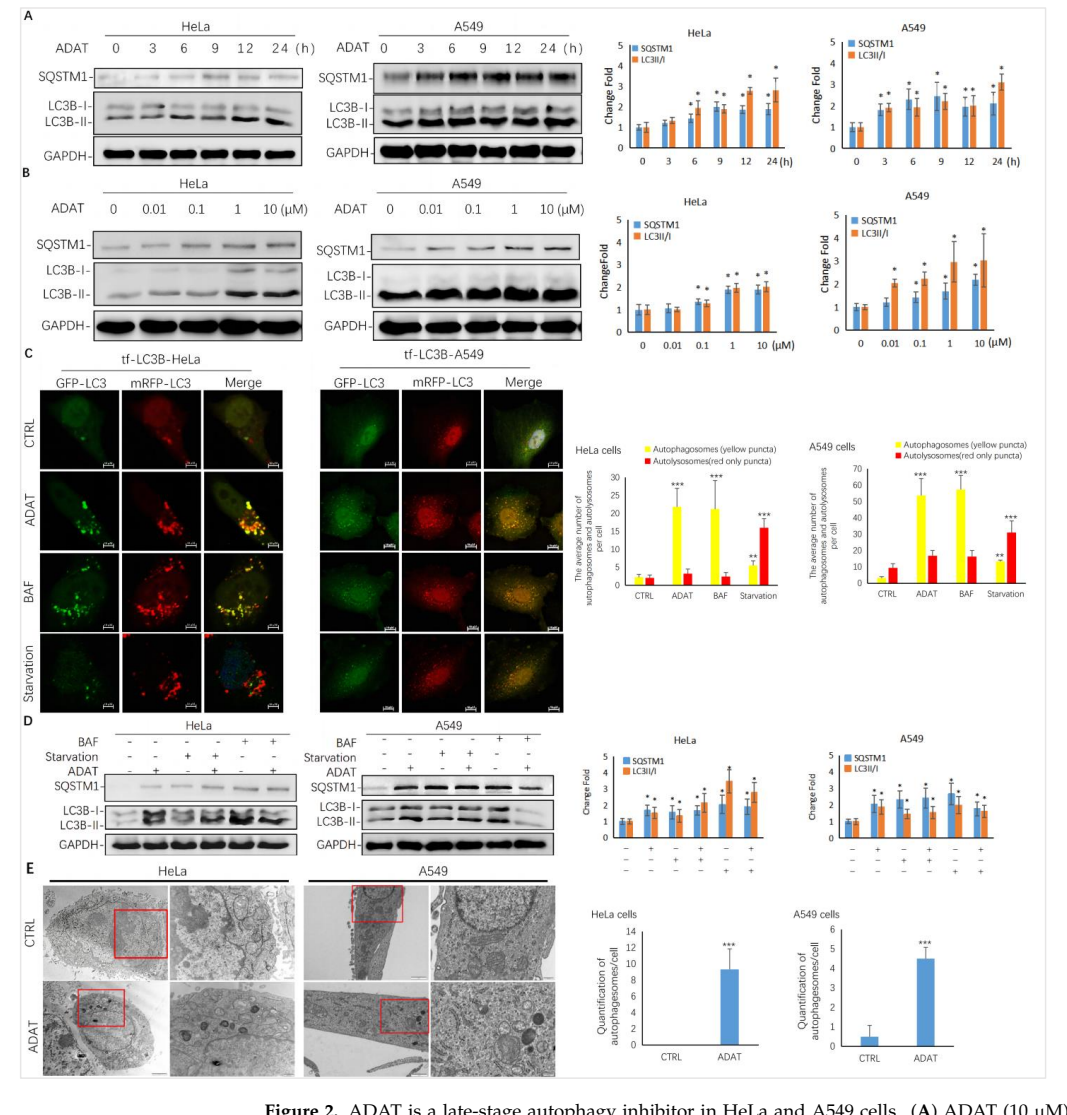
ADAT is a late-stage autophagy inhibitor in HeLa and A549 cells. (**A**) ADAT (10 μM) induced the accumulation of both LC3B-II and SQSTM1 in a time-dependent manner. (**B**) Treatment of cells with ADAT for 12 h induced the accumulation of both LC3B-II and SQSTM1 in a concentration-dependent manner. (**C**) ADAT (10 μM) or BAF (25 nM) significantly increased LC3B-II yellow puncta, but not LC3B-II red-only puncta, in mRFP-GFP-LC3-expressing cells. Scale bar = 10 μm. (**D**) BAF (25 nM) treatment for 12 h failed to further induce the accumulation of either LC3-BII or SQSTM1 in ADAT (10 μM)-treated cells when compared to control cells. Scale bar = 10 μm. (**E**) Treatment of cells with ADAT (10 μM) for 6 h induced the accumulation of double membrane autophagosomes, as shown in the electron micrographs. Scale bar = 500 nm. All experiments were independently repeated three times (*n* = 3; *, *p* < 0.05; **, *p* < 0.01; ***, *p* < 0.001 vs. control). Quantification of average number of autophagosomes and/or autolysosomes per cell in (**C**) and (**E**) is expressed as mean ± S.E., *n* ≈ 10 cells of two independent experiments.

As shown in Figure 2A,B, ADAT induced the accumulation of LC3B-II and SQSTM1/p62 in both a time- and concentration-dependent manner in HeLa or A549 cells, suggesting that it is a late-stage autophagy inhibitor. To assess whether ADAT inhibits the fusion between autophagosomes and lysosomes, thus increasing both LC3B-II and SQSTM1, we treated tf-LC3B cells with DMSO, ADAT, nutrient deficient medium, or BAF (bafilomycin A1). Nutrient deficiency induces autophagy, and BAF is an inhibitor of the vacuolar proton pump, which can inhibit late-stage autophagy by blocking the fusion of autophagosomes and lysosomes. In tf-LC3B cells, the LC3-II positive autophagosomes are labeled with both GFP and mRFP signals shown as yellow puncta, and autolysosomes are shown as red-only puncta because GFP loses its fluorescence in acidic autolysosomes [22]. As shown in Figure 2C, BAF induced the accumulation of yellow puncta and blocked autophagic flux. Similar to BAF, ADAT significantly increased the number of yellow puncta in tf-LC3B cells. In contrast, many red-only puncta were observed in nutrient-deficient tf-LC3B cells. Next, we co-incubated BAF-treated or serum-starved cells with ADAT. We showed that ADAT further increased the LC3B-II level in the starved cells, but not in the BAF-treated cells (Figure 2D). Moreover, under the electron microscope, more autophagic vesicles were observed in ADAT-treated HeLa or A549 cells when compared to control cells (Figure 2E). In summary, these results suggest that ADAT is a late-stage autophagy inhibitor, which likely inhibits autophagosome-lysosome fusion to induce the accumulation of autophagosomes and to increase levels of LC3II and SQSTM1 in treated cells.

### 2.3. ADAT Inhibits Autophagic Flux by Blocking Autophagosome-Lysosome Fusion

**Figure 3:**
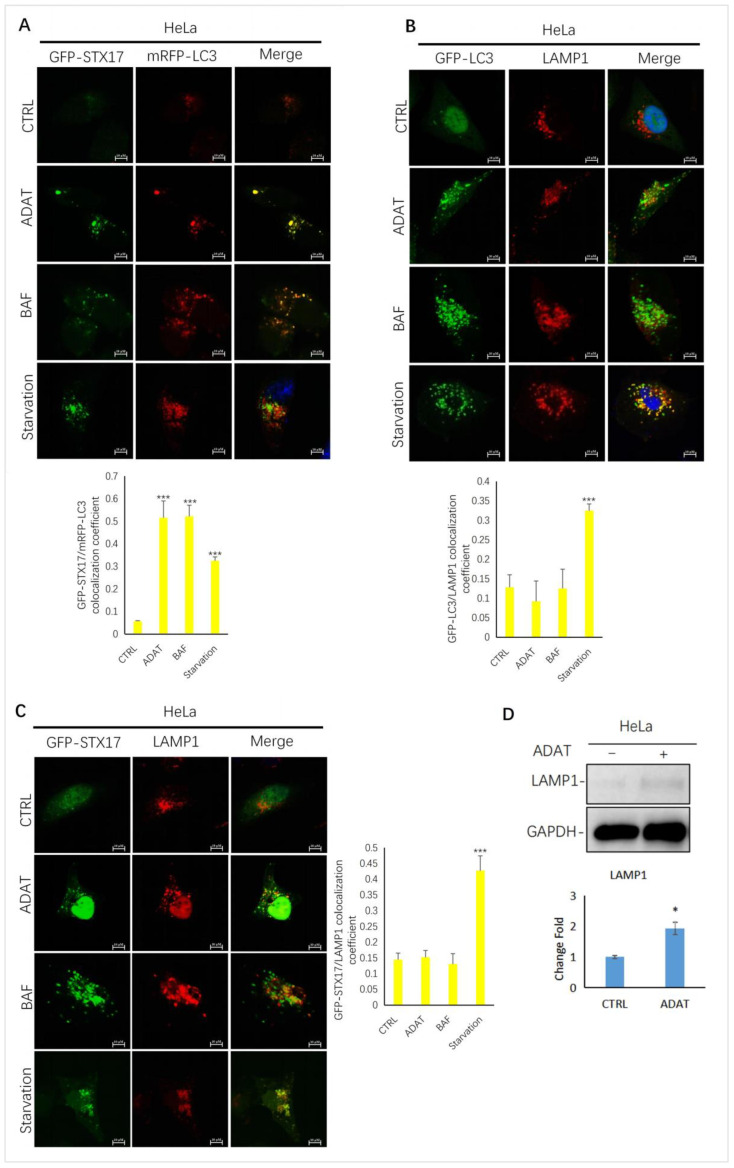
ADAT inhibits the fusion between autophagosomes and lysosomes in HeLa cells. (**A**) HeLa cells were transfected with GFP-STX17 and mRFP-LC3 and were then treated with or without ADAT (10 μM) and BAF (25 nM), or subjected to FBS starvation for 6 h, followed by confocal imaging. (**B**) HeLa cells were transfected with GFP-LC3 and were then treated with or without ADAT (10 μM) or BAF (25 nM) or subjected to FBS starvation for 6 h, followed by LAMP1 immunostaining and confocal imaging. (**C**) HeLa cells were transfected with GFP-STX17 and were then treated with or without ADAT (10 μM) or BAF (25 nM) or subjected to FBS starvation for 6 h, followed by LAMP1 immunostaining and confocal imaging. (**D**) Western blot analysis of LAMP1 levels in HeLa cells treated with DMSO or ADAT (10 μM) for 6 h. Quantification of colocalization efficiency of GFP-STX17/mRFP-LC3, GFP-LC3/LAMP1 and GFP-STX17/LAMP1 in (**A**,**C**,**D**) is expressed as mean ± S.E., n ≈ 10 cells of two independent experiments (*, *p* < 0.05; ***, *p* < 0.001, vs. control).

To further confirm ADAT-induced LC3B-II puncta representing autophagosomes, we assessed the colocalization between LC3B-II and STX17 (a SNARE protein of the autophagosome involved in autophagy [23]) in HeLa cells treated with or without ADAT. We showed that LC3B-II puncta and STX17 puncta partially co-located in the starved cells. Similar to BAF, LC3-II puncta in cells were strongly co-located with STX17 in ADAT-treated cells (Figure 3A). Next, we examined the colocalization between GFP-LC3 and LAMP1 (a lysosomal marker) in HeLa cells treated with or without ADAT. We showed that both ADAT- and BAF-induced LC3B-II puncta were weakly colocalized with LAMP1, while starvation significantly induced colocalization of LC3B-II and LAMP1 (Figure 3B). Last, we assessed the colocalization of STX17 and LAMP1 in HeLa cells treated with or without ADAT. As expected, only a few co-located yellow puncta were observed in ADAT- or BAF-treated cells, whereas the colocalization of STX17 and LAMP1 was more abundant in the starvation group (Figure 3C). Interestingly, ADAT increased the LAMP1 expression in HeLa cells (Figure 3D). Nevertheless, these results indicate that ADAT inhibits autophagosome-lysosome fusion resulting in the accumulation of autophagosomes.

### 2.4. ADAT Increasing Lysosomal pH

**Figure 4:**
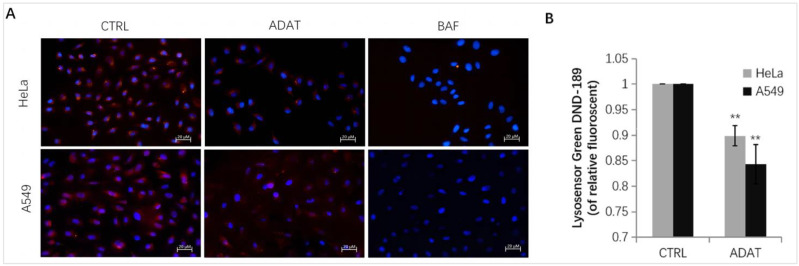
ADAT increases lysosomal pH in HeLa or A549 cells. (**A**) Fluorescence photographs of lysoTracker Red in HeLa or A549 cells treated with DMSO, ADAT (10 μM), or BAF (100 nM) for 6 h. Scale bar = 20 μm. (**B**) Treatment of cells with ADAT (10 μM) for 6 h induced an increase of lysosomal pH in HeLa or A549 cells as determined by microplate reader measurement of Lysosensor DND-189 stained cells (*n* = 3; **, *p* < 0.01 vs. control).

Late-stage autophagy inhibitors might block autophagosome-lysosome fusion or impair autolysosome degradation. Since the increase in lysosomal pH can inhibit autophagosome-lysosome fusion or/and compromise lysosome activity, we assessed whether ADAT affects lysosomal pH in HeLa or A549 cells. Fluorescent dye, e.g., LysoSensor™ Green DND-189 or LysoTracker Red, is commonly used for lysosome acidification assessment. Both dyes are sensitive to pH alteration and can be used to label and track acidic organelles (such as autolysosomes) in live cells [24,25]. The fluorescent intensity of LysoSensor™ Green DND-189 or LysoTracker Red is inversely proportional to the pH value of autolysosome. As expected, BAF treatment wrecked the acidic environment in autolysosomes, and an extremely low red fluorescent intensity was observed after the LysoTracker Red staining in BAF-treated cells (Figure 4A). Similar to BAF, ADAT significantly reduced the fluorescent intensity of LysoTracker Red or LysoSensor™ Green DND-189 (Figure 4A,B). Therefore, these results suggest that ADAT disrupts autophagic flux likely by alkalizing the lysosomal pH.

### 2.5. ADAT Might Inhibit Autophagy via Activating the mTOR/p70S6K/FOXO3a Pathway

**Figure 5:**
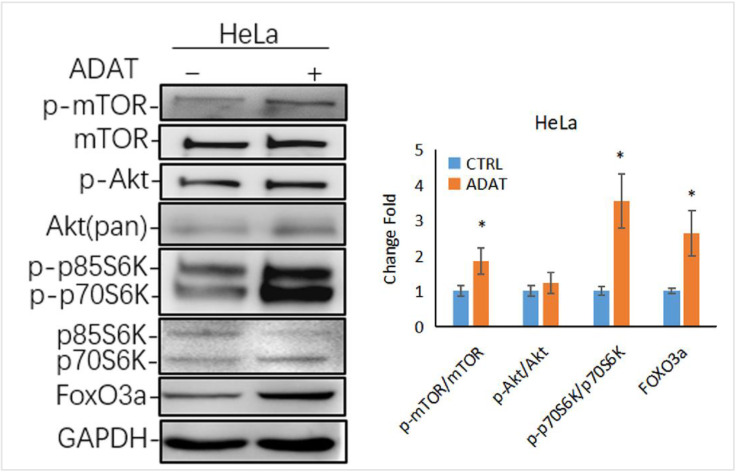
ADAT activates the mTOR/p70S6K/FOXO3a signaling pathway. Western blot analysis of p-p70S6K, FOXO3a levels in HeLa cells treated with DMSO or ADAT (10 μM) for 6 h. GAPDH was used as housekeeping gene to measure the phosphorylation degree of the corresponding protein by the ratio of p-mTOR/mTOR, p-Akt/Akt, p-p70S6K/p70S6K and the protein level of FOXO3a (*n* = 3; *, *p* < 0.05 vs. control).

Autophagy is tightly regulated by a series of signaling regulators. Among these, the mTOR/p70S6K (p70 ribosomal protein S6 kinase) signaling pathway negatively regulates autophagy induction. The induction of autophagy is associated with the inhibition of the PI3K/Akt/mTOR/p70s6k signaling pathway [26]. Interestingly, as a late-stage inhibitor of autophagy, ADAT also activated the mTOR signaling, manifested by the increased levels of phosphorylated mTOR and phosphorylated p70S6K (one mTOR target) in ADAT-treated HeLa cells when compared to control cells (Figure 5). Of note, the transcription factor FOXO3 (Forkhead box O3), an autophagy regulator and important downstream factor in the PI3K/Akt/mTOR/p70s6k pathway, is related to the basal autophagy. Inhibition of autophagy has been shown to induce FOXO3a accumulation in drug-resistant osteosarcoma cells [27], while the autophagy inhibitor BAF increased the exogenous expression of FOXO3a [28]. Consistently, ADAT increased the protein level of FOXO3a in HeLa cells (Figure 5). These results suggest that besides inhibiting autophagosome-lysosome fusion, ADAT might activate the mTOR/p70S6 pathway to inhibit the induction of autophagy.

## 3. Discussion

ADAT has been shown to be a potent PKC activator [20]. Regulation of PKC activity has been recognized as an important therapeutic strategy for various human cancers [29]. Moreover, targeting autophagy is becoming a new strategy for cancer therapy [30]. A growing number of studies have shown that cell survival/cell death effects are relevant to the tumor suppressor role of autophagy. Preclinical and clinical studies showed that autophagy plays an important role in the survival of tumor cells in the treatment of tumor radiotherapy and chemotherapy, and inhibition of autophagy improves the sensitivity of tumors to treatment [31]. Notably, preclinical and clinical trials are being used to evaluate the combination of autophagy inhibitors such as chloroquine (CQ) or hydroxychloroquine (HCQ) with chemotherapy or other reagents for a variety of cancers [32,33]. Here, we found that apoptosis was not the primary function mediating ADAT-suppressing cell growth (Figure 1C,D), but ADAT potently inhibited autophagy (Figure 2 and Figure 3). Compared to other known autophagy inhibitors, ADAT (10 μM) showed little cell toxicity (Figure 1B) and potent inhibition of autophagy, which makes it a promising anticancer agent with therapeutic potential.

By combining multiple approaches, we demonstrated that ADAT is a late-stage autophagy inhibitor that disturbs the fusion of autophagosomes with lysosomes (Figure 2 and Figure 3). Many late-stage autophagy inhibitors have been found to affect the pH or hydrolytic function of lysosomes [34,35]. To elucidate the underlying mechanism of ADAT on autophagy inhibition, we assessed whether ADAT affects lysosomal pH or lysosomal activity. Data from acidophilic dye staining suggested that ADAT alkalized the pH of lysosomes in these cells (Figure 4). The level of LAMP1 was increased (Figure 3D) and the colocalization of endogenous LC3B with LAMP1 was impeded after ADAT treatment (Figure 3B). In addition, ADAT induced the accumulation of autophagosomes (Figure 2C,E). Therefore, we identified ADAT as a late-stage autophagy inhibitor that was associated with the impairment of lysosomal functions.

The mTOR signaling plays a crucial role in regulating autophagy. mTOR forms two distinct signaling complexes, mTOR complex 1 (mTORC1) and mTORC2, by binding with different companion proteins, wherein mTORC1 maintains nutrient homeostasis through lysosomal biogenesis and autophagic processes. Under starvation and cell stress, mTORC1 activity is inhibited, resulting in the activation of the ULK1/2 complexes. The active ULK1/2 complexes are then transferred to the isolated membrane of the endoplasmic reticulum or other organelles to induce autophagy [36]. We showed that ADAT activated the mTOR/p70S6K/FOXO3a pathway by phosphorylating mTOR and p70S6K (Figure 5), suggesting that ADAT might inhibit autophagy induction by activating mTOR. Notably, ADAT-induced LC3II level was lower than that induced by BAF, and ADAT was found to decrease the LC3B-II level in the BAF-treated cells (Figure 2D). The decreased LC3-II levels in ADAT-BAF-treated cells, when compared to BAF treatment alone, is likely due to ADAT-mediated inhibition of autophagy induction. Taken together, ADAT is a dichotomic autophagy inhibitor targeting both early- and late-stage autophagy.

The activation of the class I phosphoinositide 3-kinase (PI3K)/Akt/mTOR/p70S6K pathway inhibits the induction of autophagy. The PI3K/AKT/mTOR/p70S6K signaling pathway is the primary pathway that regulates autophagy under certain conditions, such as starvation, oxidative stress, infection, and tumor suppression [4,37]. Although ADAT failed to affect the PI3K or AKT, it significantly induced the phosphorylation of mTOR and p70S6K (Figure 5). In recent years, emerging evidence demonstrated the importance of transcriptional regulation (like TP53, FOXOs, STAT3, etc.) of autophagy. FOXO, a transcription factor that regulates autophagy, is regulated by autophagic degradation. FOXO3 may act as a cell surveillance mechanism to maintain autophagy homeostasis and is targeted for degradation by basal autophagy [38]. Research has found that the proteasome degrades FOXO3a in response to various signaling events [39]. In addition, FOXO3a regulates the basal rate of autophagy. It was found that basal FOXO3a turnover can occur via the lysosome, and BAF or chloroquine treatment increased the amount of FOXO3a protein [28]. Jiang et al. showed that the inhibition of autophagy results in the increased expression of FOXO3a in cisplatin-resistant cancer cells [27]. Here we showed that ADAT exhibited a similar effect on FOXO3a. Yet, it remains to be determined whether ADAT-induced FoxO3a is due to its effects on activating mTOR signaling or inhibiting autophagosome-lysosome fusion. Also, whether ADAT induces FOXO3a overexpression to compromise lysosome function remains to be determined.

## 4. Materials and Methods

### 4.1. Materials and Antibodies

The ADAT was isolated from *Lyngbya majuscula*, which was collected from Xiao-Liuqiu Island of Taiwan. The purity of ADAT was proved by ^1^H and ^13^C NMR spectra (Figures S1 and S2). The compound was dissolved in dimethyl sulfoxide (DMSO, D8418, Sigma) to make a stock solution at 100 mM, which was stored at −20 °C and diluted with proper medium to various concentrations before use. HeLa cells (CCL-2™) (human cervical cancer cells) were purchased from American Type Culture Collection (ATCC). A549 cells (human alveolar adenocarcinoma basal epithelial cells) were kindly provided by Stem Cell Bank, Chinese Academy of Sciences. Fetal bovine serum (FBS, SA211.02) for cell culture was purchased from CellMax. Dulbecco’s modified Eagle medium (DMEM, C11995500), F12-K medium (21127022), penicillin/streptomycin (15140122), PBS (C20012500), 0.25% Trypsin-EDTA (25200072), Hanks’ Balanced Salt Solution with calcium (HBSS, 14025-092), and calcium-free HBSS (C14175500BT) were purchased from Gibco.

The primary antibodies against SQSTM1 (#39749), Beclin1 (#3495), BCL-2 (#15071), LAMP1 (#9091), ATG5 (#12994), PI3KC3 (#4263), Caspase-3 (#9662), Cleaved Caspase-3 (#9661), Phospho-mTOR (Ser2481) (#2974), mTOR (#2983), Phospho-Akt (Ser473) (#4060), Akt (pan) (#4691), Phospho-p70S6 Kinase (Thr389) (#9205), p70S6 Kinase (#2708), and GAPDH (#2118) were purchased from Cell Signaling Technology. The primary antibody against LC3B (#L7543) was purchased from Sigma-Aldrich. Anti-rabbit IgG HRP-linked antibody and anti-mouse IgG HRP-linked antibody were purchased from Cell Signaling Technology.

LV-GFP-PURO, LV-h-LC3-GFP-PURO, LV-mcherry-BSD, and LV-h-LAMP1 + mcherry-3xflag-BSD were purchased from Hanbio Biotechnolagy. Puromycin 2HCl (S7417) was purchased from Selleck. Pierce™ BCA Protein Assay Kit (23225) and RIPA Lysis and Extraction Buffer (89900) were purchased from Thermo Fisher. Immobilon^®^-PSQ Transfer Membrane (0.2 μM, ISEQ00010) and Bafolimycin A1 (#196000) were purchased from Merck Millipore. ProLong™ Diamond Antifade Mountant with DAPI (#P36962) was purchased from Invitrogen. BBcellProbe^®^ F07 Intracellular Ca^2+^ Assay Kit (#BB-44129) was purchased from BestBio. LysoSensor™ Green DND-189 was purchased from Dalian Meilun Biotechnology. LysoTracker Red was purchased from Beyotime.

### 4.2. Cell Culture

HeLa or A549 cells were maintained in DMEM or F-12K medium, respectively, plus 10% FBS and supplemented with 100 U·mL^−1^ penicillin and 100 mg·mL^−1^ streptomycin in a humidified incubator at 37 °C with 5% CO_2_.

### 4.3. Cell Viability Assay

Cells were cultured in 96-well plates at 3 × 10^3^ cells per well. After pretreatment with ADAT for 24 h, cells were incubated with 10% (*v*/*v*) MTT solution in regular medium for 4 h before removing solution. Then, 150 μL of DMSO solution was added to each well, and the OD value was read at 570 nm using a Cytation 5 Imaging Reader (BioTek, Winooski, VT, USA).

### 4.4. DAPI Staining

Cells were plated on the round coverslip (14 mm) within 24-well plates. After pretreatment with ADAT for 24 h, cells were fixed in 4% paraformaldehyde for 20 min, then stained with DAPI solution for 10 min in the dark and fixed on the glass slide. Fluorescence photographs were taken using the Cytation1 Imaging Reader (BioTek, USA).

### 4.5. Confocal Microscopy Imaging

Cells were plated on the round coverslip (14 mm) within 24-well plates. After treatment of DMSO, ADAT, rapamycin, or Bafolimycin A1, cells were fixed in 4% paraformaldehyde for 20 min at 4 °C and then dyed and fixed on the glass slide using ProLong™ Diamond Antifade Mountant with DAPI. Fluorescence photographs were taken using a confocal laser-scanning microscope (Nikon A1 HD25). Different fields of view were analyzed on the confocal laser-scanning microscope for each labeling condition, and representative results were shown.

### 4.6. Transmission Electron Microscopy

Transmission electron microscopy was used to visualize the occurrence of autophagy evaluated by autophagosome formation. Briefly, HeLa cells were incubated with DMSO or ADAT for 6 h. After treatment, cells were fixed in 2.5% glutaraldehyde for 1 h, collected, and transferred to centrifuge tubes using cell scrapers. Subsequently, cells were centrifuged at 800 rpm·min^−1^ for 5 min and transferred to EP tubes with new fixation fluid for further fixation at 4 °C for 2 h, followed by post-fixed with 1% osmium tetroxide for 1–2 h. After being dehydrated in different concentrations of alcohol (50%, 70%, 90%), 90% alcohol: 90% acetone (1:1), and acetone (90%, 100%), samples were clear in propylene oxide and then embedded in epon. Embedded specimens were kept at 70 °C oven overnight and double stained with uranyl acetate and Reynolds lead citrate for 10 min. Photos were taken under the transmission electron microscope (Hitachi HT7800, Tokyo, Japan).

### 4.7. Lysosomal pH Assessment

LysoSensor™ Green DND-189 or LysoTracker Red was used to measure the pH value of lysosomes according to the manufacturer’s protocols, which become more fluorescent in acidic environments. Briefly, after treatment, cells were loaded with 2 μM LysoSensor™ Green DND-189 or 50 nM LysoTracker Red in prewarmed HBSS for 30 min at 37 °C. After washing with PBS twice, the fluorescence intensity of LysoSensor™ Green DND-189-labeled cells was immediately analyzed (Ex/Em: 485/530 nm). Fluorescence photographs were taken using the Cytation1 Imaging Reader (BioTek, USA).

### 4.8. Western Blot Analysis

Cells were washed with PBS twice and then lysed with ice-cold protein lysis buffer after 6 h pretreatment of ADAT. The lysates were homogenized by an Ultrasonic Processor (Sonics Vibra Cell™) for 30 s and centrifuged at 13,000 rpm for 15 min at 4 °C to remove debris. After concentration measurement and denaturation by 5 × SDS loading buffer at 95 °C for 10 min, 10–30 μg protein per sample was separated by 4–20% Mini-PROTEAN TGX^®^ Gels (Bio-rad) according to the different molecular weights of the proteins. After electrophoresis, proteins were transferred to the PVDF membrane (Millpore), and the membranes were blocked with 5% skim milk in TBST with 0.1% Tween-20 for 1 h at room temperature. The membrane was incubated with appropriate primary antibodies, and the immunoreactive bands were developed with ECL Western blot substrate (Beyotime, P0018FM).

### 4.9. Statistical Analysis

All experiments were performed in duplicate and repeated at least three times. Results were presented as mean ± standard error of the mean (SEM). The statistical significance of differences was determined by one-way ANOVA or the Student’s *t*-test with SPSS (version.17.0). *p* < 0.05 was considered significant.

## 5. Conclusions

ADAT (10 μM) had little effect on apoptosis, but alkalinized lysosomal pH and inhibited autophagosome-lysosome fusion, resulting in the accumulation of autophagosomes. Meanwhile, ADAT (10 μM) activated the mTOR/p70S6K signaling pathway, likely leading to the inhibition of autophagy induction. Taken together, ADAT is a dichotomic autophagy inhibitor that inhibits both late-stage (autophagosome-lysosome fusion) and early-stage (autophagy induction) autophagy.

## Data Availability

Not applicable.

## Supplementary Materials

The following supporting information can be downloaded at: https://www.mdpi.com/article/10.3390/md21010046/s1, Figure S1: ^1^H spectrum of anhydrodebromoaplysiatoxin; Figure S2: ^13^C NMR spectrum of anhydrodebromoaplysiatoxin.
