# Supplementary material for: Identification of Anhydrodebromoaplysiatoxin as a Dichotomic Autophagy Inhibitor
Source: Mar Drugs. 2023 Jan 10;21(1):46. doi: 10.3390/md21010046 (PMC9862050; doi:10.3390/md21010046)
Supplement: Supplementary file 1 [file marinedrugs-21-00046-s001.zip › marinedrugs-2079675-supplementary.pdf]

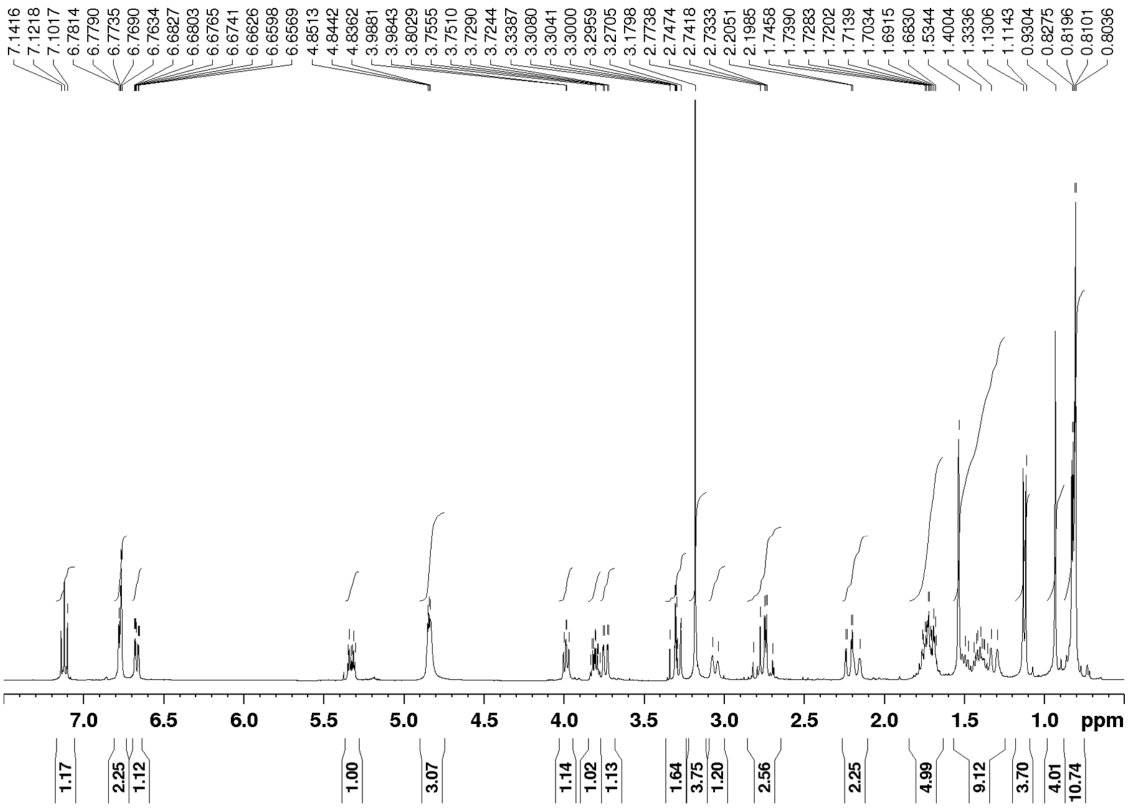

Figure S1: ^{1}H spectrum of anhydrodebromoaplysiatoxin

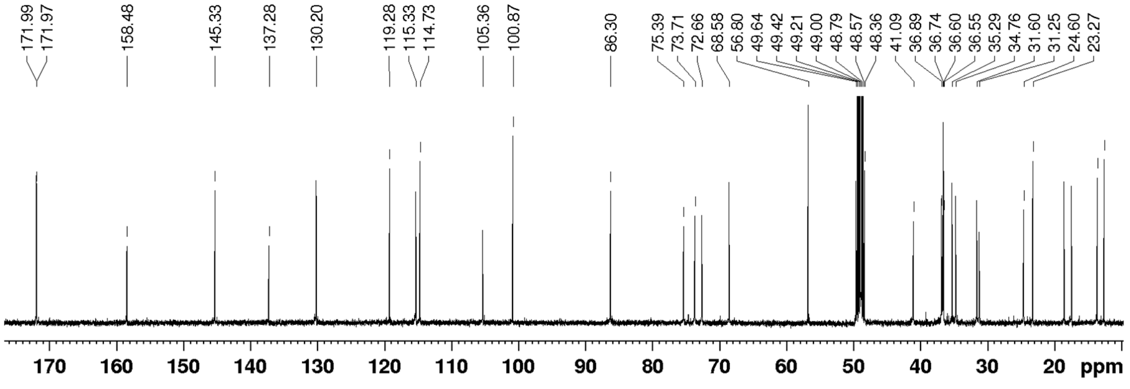

Figure S2: ^{13}C NMR spectrum of anhydrodebromoaplysiatoxin
